# Coding Region Mutation Screening in Optineurin in Chinese Normal-Tension Glaucoma Patients

**DOI:** 10.1155/2019/5820537

**Published:** 2019-05-06

**Authors:** Jing Na He, Shiyao Lu, Li Jia Chen, Pancy Oi Sin Tam, Bi Ning Zhang, Christopher Kai Shun Leung, Chi Pui Pang, Clement Chee Yung Tham, Wai Kit Chu

**Affiliations:** Department of Ophthalmology & Visual Sciences, The Chinese University of Hong Kong, Hong Kong

## Abstract

**Purpose:**

To study the roles of sequence alterations in the optineurin (*OPTN*) gene-coding region in normal-tension glaucoma (NTG) among Chinese patients.

**Methods:**

Genomic DNA was extracted from 190 NTG patients and 201 control subjects. The thirteen exons of *OPTN* were amplified by polymerase chain reaction and analyzed by direct sequencing. Detected sequence changes were compared between NTG patients and control subjects.

**Results:**

Seven sequence changes in *OPTN* were identified in both NTG patients and control subjects. Among them, c.464G>A (T34 T), c.509C>T (T49T), c.806G>A (V148V), and c.959T>C (P199P) were synonymous codon changes, whilst c.655T>A (M98K), c.1996G>A (R545Q), and c.1582T>C (I407T) were missense changes. Two previously reported heterozygous mutations, c.458G>A (E50K) in exon 4 and c.691_692insAG in exon 6, were not found in this study. Out of these seven *OPTN* sequence variants, c.464G>A (T34T) was significantly associated with NTG in both the allelic and genotypic association analyses (allelic association: *p* = 0.0001, OR = 2.20, 95% CI: 1.46-3.31; genotypic association: *p* = 0.0001), whereas the association of other variants with NTG did not reach statistical significance (*p* > 0.05). Variants c.1582 T>C (I407T) and c.806G>A (V148V) were identified in one and two NTG patients, respectively, but not in the control subjects.

**Conclusions:**

This study confirmed the association of the OPTN T34T variant with NTG, suggesting that *OPTN* is a susceptibility gene for NTG in Chinese. Moreover, a variant with amino acid change (I407T) was identified in NTG but not in controls. Further studies are warranted to assess whether this variant is a causative mutation for NTG.

## 1. Introduction

Glaucoma, a leading cause of irreversible blindness worldwide [1], is a heterogeneous group of optic neuropathies characterized by progressive degeneration of the optic nerve, and it could be detected clinically as cupping of the nerve head, thinning of the retinal nerve fiber layer, and typical visual field defect [2]. According to the morphology of the anterior chamber angle, primary glaucoma can be classified into primary open-angle glaucoma (POAG) and primary angle-closure glaucoma (PACG). According to the highest intraocular pressure (IOP), POAG has been conventionally divided into high-tension POAG (HTG, IOP > 21 mmHg) and normal-tension POAG (NTG, IOP ≤ 21 mmHg).

Currently, the molecular basis of NTG is not fully understood. Previous studies have reported gene mutations in NTG patients, like endothelin-1 (*EDN1*), optic atrophy type 1 (*OPA1*), and optineurin (*OPTN*) [3–5]. In 2002, Rezaie et al. identified *OPTN* mutations that were responsible for some of the hereditary NTGs [5]. There are 16 exons in the *OPTN* gene. Exons 1-3 are noncoding sequence, and exons 4-16 code for a 577-amino acid protein. Four mutations in *OPTN*, c.458G>A (E50K), c.655T>A (M98K), c.1996G>A (R545Q), and c.691_692insAG (2 bp “AG” insertion) were detected from 54 families with adult-onset POAG in which most families displayed normal IOP [5]. Subsequently, disease-associated variants have been found by other investigators. One of the rare mutations, E50K, showed a strong association with POAG, particularly with NTG [6].

The prevalence of NTG in Asia is higher compared that in Caucasian and African populations [7]. In China, the proportion of NTG in POAG was as high as 85% in Guangzhou, a city in southern China, and 90% in the Handan Eye study in northern China [8, 9]. It is important to identify gene mutations and genetic association with NTG in Asians. In this study, we sought to identify *OPTN* coding sequence alterations in Chinese population and their association with NTG in order to compare findings from other populations and to identify novel *OPTN* associations in NTG.

## 2. Subjects and Methods

### 2.1. Case and Control Study Subjects

Unrelated patients with NTG were recruited from the Eye Centre, Prince of Wales Hospital, and Hong Kong Eye Hospital, Hong Kong. The study protocol was approved by the Ethics Committee for Human Research of the Chinese University of Hong Kong and adhered to the tenets of the Declaration of Helsinki. Informed consents were obtained from all study subjects. Diagnosis was based on meeting all the following criteria: exclusion of secondary cause (steroid-induced glaucoma, neovascular glaucoma, uveitis, or trauma); anterior chamber angle open (grades III or IV on gonioscopy); characteristic optic disc changes (vertical cup-to-disc ratio is >0.5, disc hemorrhage, or thin/notched neuroretinal rim); and characteristic visual field changes with reference to Anderson's criteria for minimal abnormality in glaucoma [10]. Visual field was evaluated by a perimeter (Humphrey Field Analyzer; Carl Zeiss Meditec, Dublin, CA, USA) using the Glaucoma Hemifield Test. Central corneal thickness (CCT) was also measured. The mean of 5 CCT readings was 538.1 *μ*m ± 36.5 *μ*m for right eyes and 539.1 ± 36.8 *μ*m for left eyes (mean ± standard deviation). Several cross-sectional studies have indicated a range of normal CCT between 537 and 550 *μ*m in which the IOP measurements could be considered as accurate [11, 12]. Therefore, the CCT readings of our cohort were within the normal range which the IOP measurements should be accurate. To categorize NTG, multiple IOP measurements were done by using applanation tonometry, with no more than one reading equal to or higher than 21 mmHg when no IOP-lowering drugs were used. We have recorded the histories of the following systemic conditions: systemic hypertension, ischemic heart disease, diabetes mellitus, hypercholesterolemia, cerebrovascular accident, and cancer. Also, the use of IOP-lowering drugs and IOP-lowering surgeries were also recorded. These records were used for the exclusive diagnosis of NTG. Patients with congenital glaucoma or family history of glaucoma were also excluded. 190 NTG patients were included in this study. People who attended the clinic for conditions other than glaucoma, including mild senile cataract, floaters, mild refractive errors, and itchy eyes were recruited as unrelated control subjects. There were 201 subjects in the control group.

### 2.2. Polymerase Chain Reaction

Genomic DNA was extracted from whole blood by the QIAamp Blood Kit (Qiagen, Hilden, Germany). 13 pairs of PCR and sequencing primers were designed according to all coding sequence of *OPTN*, including intron-exon boundaries (Table 1). PCR was performed on a PCR machine (C1000 Touch, Bio-Rad, Hercules, CA, USA), with an initial denaturation step of 5 minutes at 95°C, followed by 45 cycles of 95°C for 30 seconds, annealing temperature of 55–62°C for 30 seconds, 72°C for 1 minute, and a final extension step of 72°C for 5 minutes. Each 25 *μ*L reaction contained 1.5–2.5 mM MgCl_2_, 1 U *Tag* polymerase (Ampli*Tag* Gold, Biosystems, Foster City, CA, USA), 200 *μ*M dNTPs, 1 *μ*L genomic DNA, and 400 pM primers. Agarose gel electrophoresis was performed after PCR to confirm the amplification efficiency.

### 2.3. DNA Sequencing

PCR products were sequenced using a cost-saving protocol on an automated DNA sequencer (model: ABI 377XL; Applied Biosystems, Foster City, CA, USA) [13]. Sequence data were aligned with sequence-analysis software (BioEdit, version 7.0.5.3, Carlsbad, CA, USA) and compared with the published *OPTN* gene sequence (Ensemble transcript ID: ENST00000378748.7, provided in the public domain by European Molecular Biology Laboratory's European Bioinformatics Institute, Hinxton, Cambridge, UK; NCBI reference sequence: NM_001008211, provided in the public domain by the National Center for Biotechnology Information, Bethesda, MD, USA).

### 2.4. Statistical Analysis

The frequencies of various alleles and genotypes between NTG patients and control subjects were compared using the *χ*^2^ test or Fisher's exact test. *p* < 0.05 was considered statistically significant.

## 3. Results

### 3.1. *OPTN* Variants Detected in the Study Subjects

Seven sequence changes in *OPTN* were identified in this study: four were synonymous codon changes and three were missense changes (Table 2). We found c.655T>A (M98K), c.1996G>A (R545Q), c.464G>A (T34T), c.509C>T (T49T), and c. 959T>C (P199P) in both NTG patients and control subjects. Among these mutations, some of them were also identified as homozygotes in NTG subjects: M98K was found in 6 NTG patients and 4 controls, while T34T was observed in 9 NTG patients. Two other mutations, c.1582T>C (I407T) and c.806G>A (V148V), were identified in one and two NTG patients, respectively, but not in control subjects. I407T was found in a 48-year-old male NTG patient. Regarding the missense variants, prediction using PolyPhen and SIFT suggests these amino acid changes would not affect the protein functions.

### 3.2. Distribution of *OPTN* Variants in NTG Patients and Control Subjects

T34T was found to be significantly associated with NTG when comparing the allelic and genotypic frequencies between NTG patients and control subjects (allelic association: *p* = 0.0001, OR = 2.20, 95% CI: 1.46-3.31; genotypic association: *p* = 0.0001), whereas the association of other variants with NTG did not reach a statistical significance (*p* > 0.05).

## 4. Discussion

Optineurin is a receptor protein of autophagy. There is a LC3-interacting region (LIR) and an ubiquitin-binding domain (UBD) in OPTN, which could guide the transportation of ubiquitylated cargos into autophagosome, a specialized organelle to degrade targeted molecules. Autophagy is a dynamic process delivering cytosolic materials to lysosome for degradation which is a key process in maintaining cellular and tissue homeostasis [14]. Autophagy was reported to be associated with the pathogenesis of glaucoma [15]. The decrease of autophagic activity was observed in porcine trabecular meshwork (TM) cells which may represent the progressive failure of cellular TM function and contributes to the pathogenesis of POAG [16]. Accumulation of autophagic vacuoles and autophagosomes was also found in retinal ganglion cells (RGCs) of glaucoma animal models which may be related to the pathogenesis of glaucoma [17, 18].

Three variants found in the present study involve codon changes. M98K has been reported to be associated with POAG [5]. However, our data showed no significant difference in the prevalence between NTG patients and controls. M98K is located in the Tank-binding kinase 1 (TBK1) binding domain of OPTN. Binding between the OPTN and ubiquitin polypeptide chains could be enhanced by TBK1-mediated phosphorylation on optineurin [19]. Previous studies found that OPTN M98K could activate TBK1, which in turn enhanced the phosphorylation on Serine-177 on OPTN. The phosphorylated OPTN could enhance autophagosome formation and lead to retinal cell death [20, 21]. Previously, OPTN I407T was only found in HTG patients [22]. In this study, I407T was found in a NTG patient but not in control subjects. I407T is located in the coiled-coil region of OPTN. How I407T influences OPTN function is still not very clear. R545Q were located in exon 16 and are near the zinc finger domain within OPTN. Although it was reported in one NTG study before [5], our current study and two previous studies did not detect an association of R545Q with NTG [23, 24]. T34T was the only variant that showed significant association with NTG in our study. A meta-analysis for the M98K, T34T, and R545Q polymorphisms demonstrated a significant association only between T34T and POAG in Asians under the allele contrast and dominant models for allele A [25]. It has also been reported that T34T significantly increased the risk of NTG [26]. On the other hand, several variants reported to be associated with NTG were not detected in our study. E50K is a rare variant but is strongly associated with POAG especially NTG in Caucasians [27, 28]. 691_692insAG was reported in one Ashkenazi Jewish POAG patient from Russia [27]. However, in a Japanese cohort, E50K and 691_692insAG were not found in both POAG and NTG patients [24, 29]. We also did not detect E50K in Chinese NTG patients in this study.

Compared to previous studies of *OPTN* in NTG, we believe our cohort contains the largest number of well-defined NTG cases [6, 24, 29]. But there is still limitation that another independent cohort of Chinese NTG samples is needed to consolidate our finding. Future studies could also be improved by measuring the 24-hour IOP to better diagnose the NTG. 24-hour IOP is a powerful technique developed in recent years to monitor the changes of intraocular pressure over 24 hours. A recent review suggested that this technology could be useful for early detection of glaucoma, individualized treatment for glaucoma, improved patient adherence to glaucoma treatment, changed patient behavior, and prevention of glaucoma progression [30]. Currently, there is a commercially available 24-hour IOP device using the disposable contact lens sensor (CLS) (Sensimed Triggerfish®, Sensimed AG, Lausanne, Switzerland). Several areas remained to be further optimized in this technology. For example, its high cost could be a major issue for large-scale clinical investigations. Furthermore, this CLS technology does not measure IOP directly. Instead, the measurements are expressed in an arbitrary unit, which may not be directly comparable among individual eyes [31]. More studies are needed to further translate this technology into clinical practice.

In conclusion, the OPTN T34T was significantly associated with NTG in the Chinese population. Three reported OPTN missense variants, M98K, I407T, and R545Q, were also found in NTG patients, although the associations were not significant. Other OPTN variants, T49T, V148V, and P199P, were also not significantly associated with NTG in our cohort. Moreover, it is the first time that I407T was found exclusively in a NTG patient. Future studies are needed to confirm more OPTN mutations and dissect the pathological roles of OPTN in NTG.

## Figures and Tables

**Table 1:** Oligonucleotide primers used for PCR and sequencing of *OPTN* in NTG patients.

	Primer	Sequence (5′ to 3′)	Amplicon Size (bp)	MgCl_2_ (mM)	Anneal Temp (°C)
1	OPTN-3/4F	TTTCTGAAGCTACATATACCTTT	445	2.5	62-55 (TD)
	OPTN-3/4R	CTCACCACCAAGCCTTAGTG			
2	OPTN-5F	AGCCATGTGGTCAAGTGGAC	362	1.5	62-55 (TD)
	OPTN-5R	AAGATTCCACTGCAAATGTCAA			
3	OPTN-6F	GTGCCCAGCCTTAGTTTGATC	336	1.5	58
	OPTN-6R	AATCCTTGGCTTGTGTTGACA			
4	OPTN-7F	TTGTGTTAAATCCCTTGCATTT	202	1.5	55
	OPTN-7R	CCTCAGGTCACAACATTTGAC			
5	OPTN-8F	CAGTTCCTTTAAGCTGGTCC	381	1.5	58
	OPTN-8R	TTGCACAAGCCTGGAATATTC			
6	OPTN-9F	CCTGATCCTTTATCCCAATTGT	244	1.5	58
	OPTN-9R	GTAGTGGCCGTGGAATCTTA			
7	OPTN-10F	GGCTACTAATGGTTCAGCCTG	239	1.5	62-55 (TD)
	OPTN-10R	GGAGGATAAAATTGCTCTCTCA			
8	OPTN-11F	TGCGACGTAAAGGAGCATTG	230	1.5	62-55 (TD)
	OPTN-11R	TCGCTGCCCTTCTGACTCAA			
9	OPTN-12F	TGGGAGGCAAGACTATAAGTTT	221	1.5	55
	OPTN-12R	CGTTCAACAGTTTCTGTTCATT			
10	OPTN-13F	CAGGCAGAATTATTTCAAAAC	273	2.5	62-55 (TD)
	OPTN-13R	TTTCCAATGCGAGAATACAG			
11	OPTN-14F	CTCCTCATCGCATAAACACTGT	267	1.5	58
	OPTN-14R	GGCACCTCTTCTACGGCC			
12	OPTN-15F	ACTTCTGTGGACTGTCTGCTC	263	1.5	62-55 (TD)
	OPTN-15R	TGATTTGGAATCCATTGTAGAG			
13	OPTN-16F	TCGCCATCTGTTCTTCAAGT	272	1.5	58
	OPTN-16R	CACAAAAGCACAACTCTTGGA			

TD: touchdown PCR using a cycling program where the annealing temperature is gradually reduced by 0.2°C per cycle.

**Table 2:** *OPTN* variants observed in 190 NTG patients and 201 control subjects.

Location	Sequence change	Codon change	Allele frequency (%)		*p* value	Odds ratio	95% CI	Genotype frequency		*p* value
			NTG	Control				NTG	Control	
			*n* = 380	*n* = 402				*n* = 190	*n* = 201	
Exon 5	c.655T>A	M98K	56 (14.7)	56 (13.9)	0.75	1.07	0.72-1.59	6/44/140	4/48/149	0.76
Exon 12	c.1582T>C	I407T	1 (0.3)	0 (0.0)	0.49	N/A	N/A	0/1/189	0/0/201	0.49
Exon 16	c.1996G>A	R545Q	12 (3.2)	15 (3.7)	0.66	0.84	0.39-1.82	0/12/178	0/15/186	0.69
Exon 3/4	c.464G>A	T34T	76 (20)	41 (10.2)	0.0001	2.20	1.46-3.31	9/58/123	0/41/160	0.0001
Exon 3/4	c.509C>T	T49T	7 (1.8)	7 (1.7)	0.92	1.06	0.37-3.05	0/7/183	0/7/194	1.00
Exon 6	c.806G>A	V148V	2 (0.5)	0 (0.0)	0.24	N/A	N/A	0/2/188	0/0/201	0.24
Exon 7	c.959T>C	P199P	2 (0.5)	1 (0.2)	0.61	2.12	0.19-23.50	0/2/188	0/1/200	0.61

N/A: not applicable.

## Data Availability

Data used to support the findings of this study are included within the article.
